# Effects of Anthocyanins on Components of Metabolic Syndrome—A Review

**DOI:** 10.3390/nu16081103

**Published:** 2024-04-09

**Authors:** Michaela Godyla-Jabłoński, Ewa Raczkowska, Anna Jodkowska, Alicja Zofia Kucharska, Tomasz Sozański, Monika Bronkowska

**Affiliations:** 1 Department of Human Nutrition, Faculty of Biotechnology and Food Science, Wrocław University of Environmental and Life Sciences, Chełmońskiego 37, 51-630 Wrocław, Poland; michaela.godyla@upwr.edu.pl; 2 Department of Internal Medicine, Occupational Diseases, Hypertension and Clinical Oncology, Wrocław Medical University, Borowska 213, 50-556 Wrocław, Poland; anna.jodkowska@umw.edu.pl; 3 Department of Fruit, Vegetable and Plant Nutraceutical Technology, Wrocław University of Environmental and Life Sciences, Chełmońskiego 37, 51-630 Wrocław, Poland; alicja.kucharska@upwr.edu.pl; 4 Department of Preclinical Sciences, Pharmacology and Medical Diagnostics, Wrocław University of Science and Technology, Wybrzeże Wyspiańskiego 27, 50-370 Wrocław, Poland; tomasz.sozanski@pwr.edu.pl; 5 Institute of Health Sciences—Collegium Salutis Humanae, University of Opole, Katowicka 68, 45-060 Opole, Poland; monika.bronkowska@uni.opole.pl

**Keywords:** anthocyanins, bioavailability, metabolic syndrome, obesity, dyslipidaemia, hypertension, oxidative stress

## Abstract

Metabolic syndrome (MetS) is a significant health problem. The co-occurrence of obesity, carbohydrate metabolism disorders, hypertension and atherogenic dyslipidaemia is estimated to affect 20–30% of adults worldwide. Researchers are seeking solutions to prevent and treat the conditions related to MetS. Preventive medicine, which focuses on modifiable cardiovascular risk factors, including diet, plays a special role. A diet rich in fruits and vegetables has documented health benefits, mainly due to the polyphenolic compounds it contains. Anthocyanins represent a major group of polyphenols; they exhibit anti-atherosclerotic, antihypertensive, antithrombotic, anti-inflammatory and anticancer activities, as well as beneficial effects on endothelial function and oxidative stress. This review presents recent reports on the mechanisms involved in the protective effects of anthocyanins on the body, especially among people with MetS. It includes epidemiological data, in vivo and in vitro preclinical studies and clinical observational studies. Anthocyanins are effective, widely available compounds that can be used in both the prevention and treatment of MetS and its complications. Increased consumption of anthocyanin-rich foods may contribute to the maintenance of normal body weight and modulation of the lipid profile in adults. However, further investigation is needed to confirm the beneficial effects of anthocyanins on serum glucose levels, improvement in insulin sensitivity and reduction in systolic and diastolic blood pressure.

## 1. Introduction

According to recent data [1,2,3], there has been an epidemic increase in the incidence of non-communicable chronic diseases, which include type 2 diabetes mellitus, cardiovascular diseases, chronic respiratory diseases and cancer. They now represent the leading causes of death and disability throughout the world. With the increasing consumption of energy-dense and nutrient-poor foods, non-communicable chronic diseases, including metabolic syndrome (MetS), have become a global problem and contribute significantly to rising healthcare costs [4,5,6].

**Figure 1:**
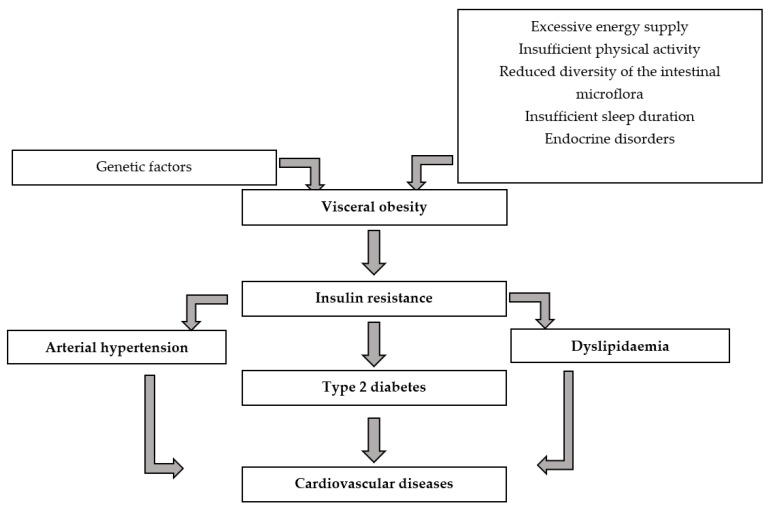
The causes and consequences of metabolic syndrome [19,20,21].

MetS refers to the co-occurrence of disorders that, when present together in the human body, increase the risk of atherosclerosis and cardiovascular disease (Figure 1). MetS-related factors include visceral obesity, dyslipidaemia (a low high-density lipoprotein cholesterol [HDLc] fraction of <40 mg/dL in men and <50 mg/dL in women, along with a triglyceride [TG] level of ≥150 mg/dL) and hypertension (systolic blood pressure [SBP] ≥ 130 mmHG and diastolic blood pressure [DBP] ≥ 85 mmHg) [7,8]. The prevalence of MetS varies from 24.0% to 69.3% among demographic and population groups [9,10].

In their systematic review and meta-analysis, Noubiap et al. [11] found that the global incidence of MetS ranged from 12.5% to 31.4%. The authors also noted a significantly higher incidence of MetS in the Americas and the Eastern Mediterranean; it correlated positively with higher country income levels. In a meta-analysis of 45,811 patients with type 1 diabetes mellitus, 23.7% of the patients had been diagnosed with MetS. The highest prevalence of MetS was observed among Australians (27.3%) and the lowest among Africans (13.1%). The incidence of MetS was slightly higher in women (25.9%) compared with men (22.5%) [12]. The incidence of MetS in Europe varies according to the country and the definition used. The lowest incidence has been reported in the United Kingdom (3.0%) and the highest in Finland (71.7%) [13]. The National Health and Nutrition Examination Survey (NHANES) 2011–2018, which involved 8183 non-pregnant women aged ≥ 20 years, led to different results. The overall prevalence of MetS increased from 37.6% in 2011 to 41.8% in 2017, mainly in participants with a low education level [14]. In addition, the prevalence of MetS has been shown to increase with age, with a 5-fold increase in women and a 2-fold increase in men [15]. Studies involving Asian populations have reported a MetS incidence of 31.0–52.8% [16,17]. In the United States, there has also been an increasing trend over the past few decades, with variations at different times. Between 1999 and 2014, the overall prevalence of MetS increased by 4.7% (from 27.6% to 32.3%) [18]. Another study conducted between 2011 and 2018 showed an increase in the prevalence of MetS from 37.6% to 41.8%. However, the initial study covering the period of 2011–2018 showed that the incidence of MetS had remained stable over this period [14]. It should be noted that the cited studies have different timeframes and cover different population groups of varying sizes, which contributes to the differences in the results obtained.

The main factors contributing to the spread of this disease are an increase in the consumption of products with a high energy density and low nutritional value and low or no physical activity. These foods are high in saturated fatty acids and simple carbohydrates and low in complete protein, polyunsaturated fatty acids, dietary fibre, vitamins, minerals and other biologically active compounds [4,5].

## 2. Criteria for the Diagnosis of MetS

The first organisation to adopt and disseminate the term ‘metabolic syndrome’ was the World Health Organization (WHO) in 1998. At that time, the WHO experts identified impaired carbohydrate metabolism, in particular decreased insulin sensitivity of target tissues, as the main criterion for the diagnosis of MetS. Additional guidelines for the diagnosis of MetS were the presence of at least two of four distinguished disorders, namely visceral obesity, dyslipidaemia, elevated BP or excretion of small amounts of protein (microalbuminuria) in the urine [22]. In 2022, several Polish societies published a position paper on the definition of MetS and practical management of patients with this condition [23]. The authors considered the presence of android obesity (waist circumference ≥ 88 cm in women and ≥102 cm in men) or body mass index (BMI) ≥ 30 kg/m^2^ and two of the three components shown in Table 1 as criteria for the diagnosis of MetS.

Overweight and obesity, especially excessive visceral fat accumulation, are major risk factors for the development of insulin resistance, type 2 diabetes mellitus, non-alcoholic steatohepatitis, ischaemic heart disease, stroke and certain types of cancer [4,5]. Researchers have shown that inappropriate diets in children and adolescents intensify the risk of diet-related diseases, including MetS, in adulthood [24,25,26,27]. There are different methods for diagnosing MetS in children and adolescents. According to the International Diabetes Federation (IDF) criteria, among children aged 10–16 years, MetS is diagnosed if there is central obesity (≥90 centile) and two of the following factors: fasting glucose ≥ 100 mg/dL or previously diagnosed type 2 diabetes mellitus, SBP ≥ 130 mmHg or DBP ≥ 85 mmHg, serum TG ≥ 150 mg/dL and high-density lipoprotein cholesterol (HDLc) < 40 mg/dL. Piotrowska et al. [28] showed that at least one component of MetS was found in approximately 15.0% of 771 students aged 10–18 years. In the study group, the most common MetS components were excessive WC, reduced HDLc, elevated serum TG and elevated BP.

An important factor that may have increased the risk of MetS in adulthood is the coronavirus disease 2019 (COVID-19) pandemic, which has caused, among other things, a change in the lifestyle of children and adolescents. This was demonstrated in a study carried out in Italy involving 965 parents and their children aged 5–18 years [29]. The authors reported an increase in the total amount of food consumed (by 50.0%), an increased intake of high-energy snacks, a decrease in physical activity and, at the same time, an increase in body weight.

Overweight and obesity during childhood and adolescence carry a number of possible risks to a person’s health and even life. They lead to numerous complications that manifest in adolescence and adulthood. Excess body fat accumulation increases the likelihood of the onset of MetS in early childhood with all of its consequences—that is, abdominal obesity, type 2 diabetes mellitus, hypertension and/or dyslipidaemia. In adults, on the other hand, it mainly increases the risk of cardiovascular disease and obesity [30,31,32].

The components of MetS have also been identified as risk factors for the development of viral infection due to their effect on the body’s immune response. This has been noted in numerous observations related to COVID-19. Indeed, there is a close association between the presence of obesity, hypertension, diabetes mellitus and cardiovascular disease and a more severe course and increased mortality among patients infected with COVID-19 [33,34,35,36].

In recent years, a great deal of research has been devoted to issues related to the prevention and effective management of MetS and non-communicable chronic diseases. Non-pharmacological management—that is, dietary changes and increased physical activity—have been shown to be the basis for the prevention and treatment of these diseases [37,38,39]. One of the documented ways to influence the prevention of MetS is the introduction of anthocyanin-rich foods; they provide numerous benefits, including the ability to counteract the development of obesity and to improve adipocyte function [40,41,42]. Epidemiological studies and nutritional interventions have demonstrated a broad spectrum of biological effects that may benefit patients with MetS-related chronic diseases [43,44].

## 3. The Occurrence and Content of Anthocyanins in Fruits and Vegetables and Their Processed Products

Anthocyanins are the largest group of water-soluble pigments belonging to the flavonoid group. They are usually responsible for the red, blue and purple colour of fruits and vegetables [45,46]. Fresh raspberry (2199.0 mg/100 g) [47], cranberry (835.2 mg/100 g) [48] and chokeberry (686.0 mg/100 g) [49] have the highest total anthocyanin contents. Other studies have indicated the following contents in whole fruit: a maximum of 22.1 mg/100 g in fresh red currants [50]; approximately 465.4 mg anthocyanins/100 g (as cyanidin-3-glucoside equivalents) in blackberries [51]; 204.3 mg anthocyanins/100 g in strawberries [52]; 287.8 mg/100 g in blackcurrants [50]; 344.9 mg/100 g in cherries [53]; and 534.2 mg/100 g in blueberries [54]. The fruit extracts with the highest anthocyanin contents include chokeberry (up to 9360.0 mg/100 g) [55], blueberry (up to about 1301.2 mg/100 g) [56] and cranberry (520.0 mg/100 g) [57]. Among vegetables, fresh red cabbage has the highest reported anthocyanin content (275.4 mg/100 g) [58]; its extracts also have the highest anthocyanin content (up to 191.4 mg/100 g) [59]. Some studies used purified extracts, such as the studies by Bushmelev et al. [55] or Roda-Serrat et al. [60], and therefore the extracts of these fruits are characterised by a higher anthocyanin content compared to fresh or frozen fruit, where the anthocyanin content of non-purified extracts is determined.

Table 2 shows the anthocyanin contents of selected fruits, vegetables and their processed products. The anthocyanin content of individual fruits and vegetables varies depending on the genotype, cultivar, growing region, weather conditions during plant growth and ripening, maturity stage at harvest, storage method of the raw material, extraction methods used, precision of the analytical methods used and the form of fruit and vegetable that was analysed [61,62,63,64,65,66]. In addition, the anthocyanin content can be expressed as total anthocyanins determined with a spectrophotometric method or as cyanidin or delphinidin derivatives determined by high-performance liquid chromatography (HPLC). Due to the large discrepancies in the results obtained by different authors, it is often difficult to compare the content of these compounds in the same raw material. The lower content of total anthocyanins in fruit products (e.g., juices) compared with fresh raw material may be due to changes in the chemical composition, pH, temperature and exposure to oxygen and light during fruit processing [67,68]. Therefore, when comparing the anthocyanin content of individual fruits and vegetables, it is important to consider the many factors that affect the final concentrations of these pigments.

## 4. Structure of Anthocyanins

The common feature of all anthocyanins is a carbon skeleton consisting of two aromatic rings linked by a three-carbon chain. There are differences among anthocyanins regarding the number of hydroxyl groups, their degree of methylation and the type and number of sugars and their site of attachment to the aglycone. The most common anthocyanin is cyanidin 3-*O*-glucoside. Anthocyanins are most commonly glycosylated with glucose in the C-3 position, but this sugar can also be rhamnose, xylose, galactose or arabinose. Many di- and trisaccharides occur in various combinations with the previously mentioned sugars. In addition to the C-3 position, other sugars can also be attached to any of the hydroxyls at the C-3′, C-5, C-5′, C-7 and even C-4′ positions. Sugars can be esterified with aromatic or aliphatic acids to produce mono- and polyacylated anthocyanins. The acids attached to the sugars in the anthocyanin molecule are usually compounds from the hydroxycinnamic acid group (ferulic, caffeic, *p*-coumaric or sinapinic acid). Such compounds are found most often in vegetables [45,46].

The chemical structure of individual anthocyanins influences their biological activity. Koss-Mikołajczyk et al. [127] showed that the number and position of methoxy and hydroxyl groups in the structure of anthocyanidins are strongly related to their antioxidant and biological activity. There was higher antioxidant activity in compounds containing more hydroxyl groups in the B ring (pelargonidin < cyanidin < delphinidin). By substituting a methoxy group in place of the hydroxyl group, there was a decrease in the antioxidant activity (malvidin < petunidin < delphinidin, peonidin < cyanidin). It has been established that for the antioxidant capacity of anthocyanins, the 3′ and 4′ hydroxyl groups in their structure, which can be easily oxidised by donating one or two electrons, play a key role [127]. The results of several studies have already indicated that delphinidin has the strongest antioxidant activity among the anthocyanidins and the high antioxidant activity of petunidin and cyanidin due to the hydroxyl groups at the 3′ and 4′ positions [128,129].

**Figure 2:**
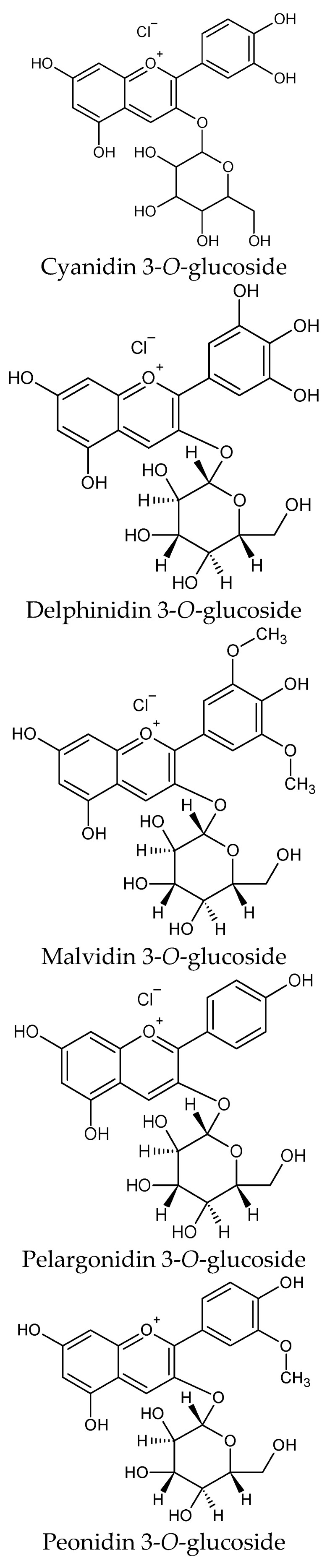

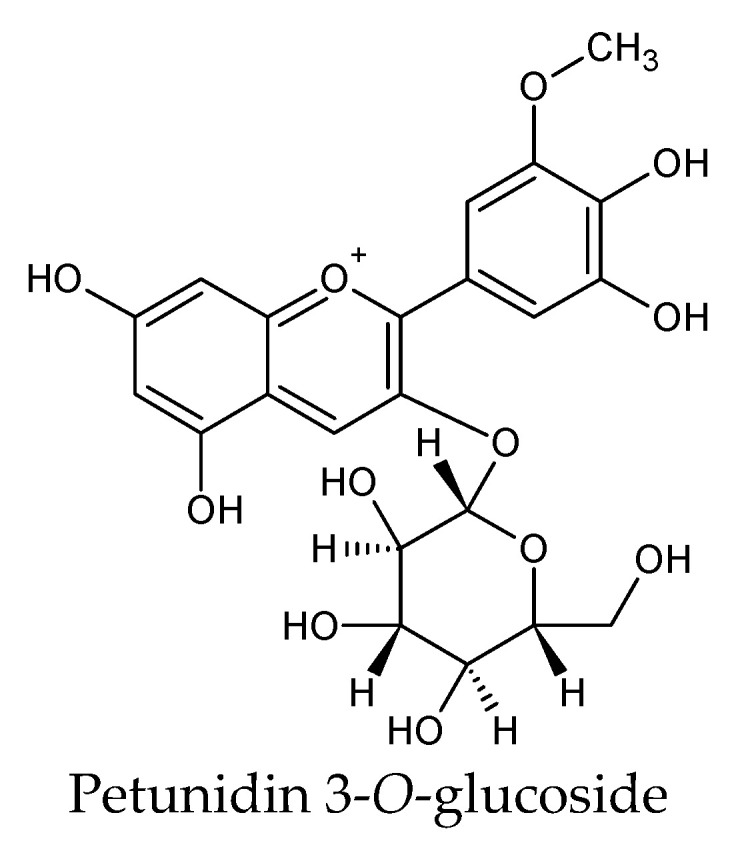
Chemical structure of some mono- or di-glycosylated anthocyanins.

Malvidin, pelargonidin and peonidin show lower reducing power because they have a single hydroxyl group in the B ring [130]. The chemical structure of some mono- or di-glycosylated anthocyanins is shown in Figure 2. In addition, the natural acylation of anthocyanins leads to more stable compounds with more beneficial biological effects compared with their non-acylated analogues [131]. Fei et al. [132] demonstrated that four acylated anthocyanins with maleic anhydride derived from blueberries via a solid-phase grafting method showed better stability than their native non-acylated forms, regardless of the degree of acylation. Their antioxidant potential, however, was somewhat lower, as they were less effective at capturing 2,2-diphenyl-1-picrylhydrazyl (DPPH) radicals. In turn, the methylation of anthocyanins plays a role in their water solubility and structural stability [133,134]. Glycosylation also improves the structural stability of anthocyanins, but it also reduces their bioactivity [135].

## 5. The Effects of Anthocyanins on the Prevention and Supportive Treatment of the Components of MetS

### 5.1. In Vitro Studies

Numerous in vitro studies have demonstrated the positive effects of anthocyanins contained in fruit extracts on the components of MetS (Table 3), including type 2 diabetes mellitus. Indeed, they have been found to inhibit carbohydrate digestive enzymes (especially α-glucosidase), to facilitate glucose transporter 4 (GLUT4) translocation, to increase glucagon-like peptide-1 (GLP-1) secretion and to interact with sodium–glucose cotransporter (SGLT) to delay glucose absorption in various organs and tissues [136,137,138]. Anthocyanins have also been shown to improve cellular glucose uptake and to enhance tissue insulin sensitivity [139,140,141].

Fruits are an excellent source of anthocyanins with anti-inflammatory properties, which may hold promise in the development of nutraceuticals for the prevention and treatment of chronic inflammation associated with MetS [142]. A study conducted on human umbilical vein endothelial cells (HUVECs) demonstrated the anti-inflammatory properties of malvidin-3-glucoside and malvidin-3-galactoside contained in blueberries. They affected monocyte chemotactic protein-1 (MCP-1), which regulated monocyte/macrophage migration and infiltration, and intercellular adhesion molecule-1 (ICAM-1) and vascular cell adhesion molecule-1 (VCAM-1) [143]. Warner et al. [144] presented similar results: anthocyanin metabolites (cyanidin-3-*O*-glucoside) reduced VCAM-1 and interleukin-6 (IL-6) production. Anthocyanins may also have a positive effect on lipid metabolism by reducing cholesterol absorption. This action involves the inhibition of pancreatic lipase, reduction in micellar cholesterol solubility and inhibition of cholesterol uptake [145,146,147]. Further investigation is needed to fully understand the effects of anthocyanins on MetS components in vitro and to determine their potential as dietary supplements or the basis for novel medications.

**Table 3:** The effects of anthocyanins on the components of metabolic syndrome (in vitro studies).

Ref.	Laboratory Model	Properties	Food or Compound	Effects
[148]	B16-F10 metastatic melanoma murine cells	Antitumour effect	Blueberry	Inhibition of B16-F10 proliferation (at concentrations of >500 μg/mL); stimulation of apoptosis; ↑ total LDH activity
[145]	Caco-2 cells	Hypocholesterolaemic effect	Black rice	↓ Cholesterol uptake
[143]	HUVECs	Anti-inflammatory effect	Blueberry	↓ MCP-1, ICAM-1 and VCAM-1 production
[139]	3T3-L1 adipocytes	Antidiabetic effect	Purple corn pericarp and pure anthocyanins	↑ Glucose uptake; activation of insulin signalling
[149]	HepG2 cells (a human liver cancer cell line)	Antidiabetic effect	Mulberry	↑ Glucose consumption, glycogen content and PEPCK and G6Pase activities; ↓ glucose production
[140]	3T3-L1 adipocytes	Regulation of glucose metabolism	Cyanidin-3- rutinoside	↑ Glucose uptake
[137]	Jejunum samples from RF/J mice; HT-29, Caco-2 and NCM460 cells (human intestinal cell lines)	Antidiabetic effect	Delphinidin	Inhibition of glucose absorption
[144]	HUVECs	Anti-inflammatory effect	13C5-cyanidin-3- glucoside	↓ VCAM-1 and IL-6 production
[147]	Caco-2 cells	Hypolipidaemic effect	Cyanidin-3- rutinoside	Inhibition of pancreatic cholesterol esterase and the formation of cholesterol micelles; ↓ cholesterol uptake
[146]	Caco-2 cells	Hypolipidaemic effect	Thai berries	Inhibition of pancreatic lipase and cholesterol esterase; bind to primary and secondary bile acids; ↓ cholesterol uptake
[150]	Osteoblast culture	Anti-osteoporotic effect	Red and yellow *Cornus mas* L. fruit	↓ TRAP activity and the number of TRAP-positive multinucleated cells
[138]	HepG2 cells	Antidiabetic effect	Blueberry	Inhibition of α-glucosidase

Abbreviations: G6Pase, glucose-6-phosphatase; HUVECs, human umbilical vein endothelial cells; ICAM-1, intercellular adhesion molecule 1; IL-6, interleukin-6; LDH, lactate dehydrogenase; MCP-1, monocyte chemotactic protein-1; PEPCK, phosphoenolpyruvate carboxykinase; TRAP, tartrate-resistant acid phosphatase; VCAM-1, vascular cell adhesion molecule 1; ↓, lowering the concentration; ↑, increase the concentration.

### 5.2. In Vivo Studies with Animal Models

Numerous in vivo studies with animal models have demonstrated the health-promoting effects of anthocyanins contained in fruits and vegetables, including benefits regarding carbohydrate and lipid metabolism and anti-inflammatory, hypotensive and weight-reducing effects. Table 4 presents the effects of anthocyanins on components of MetS based on in vivo studies in animal models. The findings suggest that the effects of anthocyanins may vary depending on the dose used, the duration of the experiment, individual differences in the absorption and metabolism of biologically active compounds and the presence of other substances.

#### 5.2.1. Effects on Carbohydrate Metabolism

Several in vivo studies have shown beneficial effects of anthocyanins on carbohydrate metabolism. These compounds may ameliorate type 2 diabetes mellitus by controlling postprandial hyperglycaemia via the inhibition of α-amylase and α-glucosidase [184], which are primary risk factors for the development of MetS. Dzydzan et al. [171] showed that anthocyanins in cornelian cherries (*Cornus mas* L.) may contribute to the alleviation of hyperglycaemia. In addition, these compounds exhibited antidiabetic and antioxidant effects by inhibiting oxidative processes involving protein and lipid modification, advanced glycation and the formation or accumulation of oxidative proteins. Furthermore, the authors observed lower blood glucose levels and a reduction in excessive thirst (polydipsia), which is one of the primary symptoms of type 1 and type 2 diabetes mellitus [170]. These compounds also contribute to the stimulation of glucagon-like peptide-1 (GLP-1) secretion and to the inhibition of dipeptidyl peptidase IV (DPP-IV) [177]. In addition, anthocyanins reduce the production of reactive oxygen species (ROS), prevent endoplasmic reticulum stress and inhibit pancreatic lipase activity, resulting in improved glycaemic control and lipidaemia and protecting the liver from insulin resistance induced by a high-fat diet [158,185]. The administration of red cabbage extract to rats with streptozotocin-induced type 1 diabetes mellitus resulted in lower serum glucose, glycated haemoglobin and foetal haemoglobin levels; improved glucose tolerance; and significantly increased serum insulin, proinsulin and C-peptide levels. At the same time, the addition of the extract improved pancreatic islet morphology by increasing the number of pancreatic β-cells [167].

#### 5.2.2. Effects on Lipid Metabolism

In vivo studies have shown that anthocyanins have positive effects on lipid metabolism. These compounds in *Aronia melanocarpa* reduced the lipid content and inflammation in 3T3-L1 adipocytes and improved the blood lipid profile and degeneration of adipose tissue cells in mice fed a high-fat diet [186]. Blue honeysuckle anthocyanins had a normalising effect on plasma TG levels and lipid atherogenicity [155]. Cornelian cherry anthocyanins counteracted elevated TG levels and atherosclerotic changes in the thoracic aorta [159]. Black chokeberry anthocyanins promoted a 16.5% reduction in total cholesterol and the proatherogenic fraction of LDLc [160]. Raspberry anthocyanins normalised the serum lipid profile [169]. Black rice anthocyanin supplementation reduced TG, total cholesterol and insulin resistance in mice with obesity induced by a high-fat diet [174]. Blueberry anthocyanin extract and malvidin alleviated oxidative stress in the liver, inhibited hyperlipidaemia and improved lipid metabolism in mice with diabetes [177]. Blackcurrant anthocyanin supplementation alleviated hyperlipidaemia and hepatic steatosis and regulated hepatic lipid metabolism [175]. Zhang et al. [168] showed that anthocyanin extracts from lingonberry (*Vaccinium vitis-idaea* L.) regulated serum cholesterol metabolism and reduced inflammatory cell infiltration and fat deposition in liver cells.

#### 5.2.3. Anti-Inflammatory Effects

Anthocyanins also exert anti-inflammatory effects. Gao et al. [187] showed that anthocyanins from purple vegetables—purple cabbage, purple sweet potato, purple corn and *Gynura bicolor*—effectively inhibited the production of pro-inflammatory factors such as nitric oxide (NO) and tumour necrosis factor alpha (TNF-α) [187]. Anthocyanins have also been shown to counteract the progression of obesity and osteoarthritis [188]. Ngamsamer et al. [189] highlighted the ability of anthocyanins to reduce markers of obesity-induced inflammation. Sozański et al. [159] added cornelian cherries to the diet of hypercholesterolaemic rats and reported a significant protective effect on diet-induced oxidative stress in the liver and a reduction in elevated serum pro-inflammatory cytokine levels. In addition, supplementing the diet of rabbits fed 1% cholesterol with loganic acid contributed to a reduction in TNF-α and IL-6, indicating an anti-inflammatory effect [162]. Other authors have reported that anthocyanins reduce C-reactive protein levels [156] and increase glutathione peroxidase (GSH-PX) and superoxide dismutase (SOD) activities in the liver [164,169]. In a model of obesity induced by an unbalanced diet, anthocyanins alleviated inflammation and oxidative stress, denoted by elevated GSH-PX and SOD activities in the liver and reduced TNF-α and IL-6 gene expression [163].

#### 5.2.4. Antioxidant Properties

There are findings in the literature supporting the antioxidant properties of anthocyanins, which play a central role in minimising the symptoms of MetS. A study conducted by Wang et al. (2014) compared the total antioxidant capacity (T-AOC) of two sources of anthocyanins, pomegranate peel extract and black bean peel extract, on oxidative stress-induced hyperglycaemia in streptozotocin-induced diabetic mice. Black bean peel extract had a slightly higher antioxidant capacity (13 U/mL T-AOC) compared to pomegranate peel extract (10 U/mL T-AOC) [180]. Anthocyanins, especially from blueberries, have shown significant antioxidant properties in animal studies. Blueberry anthocyanins have shown concentration-dependent antioxidant effects in mice, increasing total antioxidant capacity and reducing oxidative stress markers such as malondialdehyde [181]. In Wistar rats, the administration of anthocyanins led to a dose-dependent improvement in the activity of serum antioxidant enzymes such as catalase, superoxide dismutase and glutathione peroxidase [182]. Furthermore, the nano-encapsulation of anthocyanins increased their stability and bioavailability in various tissues, improving protection against oxidative damage in in vivo studies [190]. In another study in guinea pigs fed a high-cholesterol diet fortified with an 8% addition of fresh blueberries for 75 days, a reduction in oxidative stress was observed by reducing malondialdehyde concentrations [183]. The above studies indicate that anthocyanins, especially those with intense pigmentation, can be used to overcome oxidative stress damage. Although most food sources contain varying levels of these compounds, it is important to consider how the food source may affect their antioxidant capacity [191,192].

#### 5.2.5. Influence on Body Weight

A number of studies have confirmed the beneficial effects of anthocyanins on weight and BMI reduction [193]. Specifically, anthocyanin supplementation at ≤300 mg/day for 4 weeks has been shown to effectively reduce BMI and body weight [194]. Additionally, anthocyanins contained in *Lycium ruthenicum* Murray fruit counteracted obesity by inhibiting pancreatic lipase and regulating the intestinal microflora [176]. Furthermore, anthocyanin extracts from *L. ruthenicum* as well as blueberries and cranberries reduced weight gain and total body fat mass [166,176]. Mulberry aqueous extracts added to the diet (0.5–2.0%, *w*/*w*) counteracted obesity induced by a high-fat diet (0.2% cholesterol and 10% corn oil) in a hamster model of obesity [153]. Mulberry extract reduced body weight and visceral fat mass and exerted a hypolipidaemic effect. Both mulberry leaf and fruit extracts have been reported to reduce body weight and visceral fat [153,154]. Similar effects have also been observed with the administration of 100–200 mg/kg body weight anthocyanins from honeysuckle [157]; 200 mg/kg body weight anthocyanins from mulberries (by 32.7%) or cherries (by 29.6%) [163]; 200 mg/kg body weight anthocyanins from purple corn, black soybean or black rice [164]; 200 mg/kg body weight anthocyanins from raspberries [169]; and 100–300 mg/kg body weight anthocyanins from blackcurrants [161,175].

#### 5.2.6. Effects on BP

There are relatively few published animal studies that have determined the effects of anthocyanins on blood pressure (BP). Xu et al. [179] showed that chronic infusion of anthocyanins into the paraventricular nucleus of rats with salt-induced hypertension reduced BP and peripheral sympathetic nerve activity. Anthocyanins from *Hibiscus sabdariffa* reduced salt-induced hypertension in rats by inhibiting components of the renin–angiotensin–aldosterone system [178]. Yamane et al. [165] reported that supplementing a standard diet with 10% freeze-dried chokeberries reduced BP in spontaneously hypertensive rats. In addition, the administration of anthocyanin-rich pigments from purple maize, purple sweet potatoes and red radishes to spontaneously hypertensive rats reduced BP and heart rate, but not body weight, compared with rats that did not receive supplementation [151]. Herawati et al. [172] reported that 50 and 100 mg/kg body weight anthocyanins from purple sweet potatoes reduced SBP (to 116.67 ± 2.80 mmHg and 106.50 ± 1.87 mmHg, respectively).

### 5.3. In Vivo Studies with Humans

The increasing prevalence of obesity, type 2 diabetes mellitus, cardiovascular diseases and cancer has prompted a search for naturally occurring compounds in fruits, vegetables and herbs that, when included in appropriate amounts in the diet and without altering physical activity and lifestyle, could reduce the risk of particular diseases. Table 5 presents the effects of anthocyanins on components of MetS based on in vivo studies that involved humans.

#### 5.3.1. Effects on Carbohydrate Metabolism

Numerous in vivo studies involving humans have demonstrated the positive effects of anthocyanins on carbohydrate metabolism. Among other actions, anthocyanins can inhibit carbohydrate-digesting enzymes. Indeed, blue honeysuckle extract inhibited the activity of α-amylase and α-glucosidase [220]. The inhibition of carbohydrate digestive enzymes can facilitate GLUT4 translocation, suppress the efficiency of DPP-IV and prevent protein tyrosine phosphatase 1B (PTP1B) overexpression [221]. A study confirmed the beneficial effects of anthocyanins on carbohydrate metabolism in people with MetS [214]. After 4 weeks of anthocyanin supplementation (320 mg twice a day), there was a 13.3% reduction in fasting serum glucose levels in the MetS group. This supplementation also improved tissue insulin sensitivity [214]. Nolan et al. [213] reported that ad hoc and short-term supplementation with New Zealand blackcurrant extract improved insulin sensitivity and reduced postprandial glucose concentrations. Solverson et al. [222] found that mixed berry preparations (consisting of blackberries, blueberries, cranberries, raspberries and strawberries) reduced the serum insulin response. Novotny et al. [202] reported the beneficial effects of anthocyanins, proanthocyanidins and total phenols contained in low-calorie cranberry juice on glucose levels and insulin resistance [202]. During a 6-week intervention with the addition of 150 g of frozen bilberries three times per week, there was a reduction in serum glucose concentrations. There was a significant improvement in tissue insulin sensitivity (by 14%) in a group of individuals with overweight or obesity (BMI ≥ 25.0 kg/m^2^) and insulin resistance who consumed a drink containing 333 mg of strawberry and cranberry polyphenols each day [207]. During a 1-week dietary intervention with a strictly anthocyanin-free ration except for the addition of blackberry among 27 men with overweight or obesity (BMI > 25 kg/m^2^), there was a significant increase in tissue insulin sensitivity. There was a significant reduction in homeostatic model assessment for insulin resistance (HOMA-IR) scores and a significant increase in the area under the curve (AUC) for non-esterified fatty acids (NEFAs) in the study group [209]. In addition, anthocyanins in black rice extract and β-glucan from oats influenced the inhibition of starch-digesting enzymes and lowered the glycaemic index of cooked white rice [223]. In contrast, Kolehmainen et al. [224] did not observe a beneficial effect of the addition of bioactive compounds on lowering serum glucose. This may be due to methodological differences between the studies and the different sizes of the study groups.

#### 5.3.2. Effects on Lipid Metabolism

Fruit and vegetable anthocyanins can modulate TG, total cholesterol, LDLc and HDLc levels to improve the lipid profile. The effect on lipid metabolism depends on the type of anthocyanin. In one study, delphinidin-based anthocyanins, but not cyanidin- and malvidin-based anthocyanins, significantly affected TG, LDLc and HDLc levels [225]. Asgara et al. [197] introduced 50 g of cornelian cherries twice a day to the diet of 9–16-year-old children and reported a reduction in total cholesterol and LDLc and TG levels and an increase in HDLc levels compared with the baseline [197]. Habanova et al. [211] showed that the regular consumption of juice containing 50% berries (25% chokeberry, 15% blueberry and 10% cranberry) and 50% apple juice could become an important strategy to reduce the risk of developing cardiovascular disease by modulating the lipid profile (a significant increase in HDLc and a reduction in total cholesterol). Only the male subjects showed a significant reduction in total cholesterol and LDLc levels [211]. Another study showed an effect of 25 or 50 g of freeze-dried strawberries only on total cholesterol and LDLc levels among people with abdominal obesity and elevated serum lipids [199]. The same effects were observed in a group of women with overweight or obesity who were given 500 mL of commercial orange juice containing approximately 250 mg of anthocyanins each day for 12 weeks [206]. Overall, several studies have reported an increase in HDLc levels and a decrease in TG, total cholesterol and LDLc levels [200,202,204,205,208,214]. In addition, there was a reduction in albumin and a transferase involved in drug and xenobiotic detoxification (γ-glutamyltransferase) with regular consumption of 150 g of frozen bilberries (*Vaccinium myrtillus* L.) three times per week. Wright et al. [198] administered dried purple carrot providing 118.5 mg of anthocyanins and 259.2 mg of phenolic acids each day to men with obesity for 4 weeks. These anthocyanins and phenolic acids did not significantly reduce total cholesterol and LDLc levels. Moreover, they exerted an undesirable effect by lowering HDLc levels [198]. Several studies have reported the lack of a positive effect of anthocyanins on the blood lipid profile [207,212,224].

The administration of 100 mL/day of chokeberry juice (containing 1177.11 mg gallic acid equivalents) or a low dose of polyphenols (294. 28 mg gallic acid equivalents) altered plasma phospholipids fatty acids (PPFAs): there was an increase in saturated fatty acids (SFAs), especially palmitic acid (PA), and a decrease in n-6 polyunsaturated fatty acids (PUFAs), primarily linoleic acid (LA) [212]. In the human body, LA can undergo metabolic interconversion to long-chain n-3 PUFAs, including eicosapentaenoic acid (EPA) and docosahexaenoic acid (DHA). PUFAs contribute to reducing oxidative stress by modulating inflammatory processes. They also reduce the risk of developing and the severity of cardiovascular diseases and are essential components in normal brain development and cognitive function [226]. An adequate PA concentration in tissues maintains the physical properties of membranes as well as the attachment of fatty acids to specific proteins, which contribute to the hydrophobicity of proteins and to their membrane association. However, a balance between the daily ration of PA and PUFAs is necessary to maintain the balance of membrane phospholipids. Modern lifestyles based on an excessive supply of energy and simple carbohydrates and low physical activity contribute to an imbalance in the PA concentration. Excessive PA accumulation in tissues may contribute to the development of dyslipidaemia or hyperglycaemia [227].

#### 5.3.3. Anti-Inflammatory Effects

Similarly to animal models, anthocyanins exert anti-inflammatory effects in humans. According to Masheta et al. [217], anthocyanin extracts reduced oxidative stress and inflammation in people with hypertension and diabetes mellitus, confirming their potential to delay complications associated with these chronic diseases. In addition, blueberry, blackberry, cranberry and blackcurrant anthocyanins have been reported to inhibit inflammation in preadipocytes, which store low-grade inflammatory biomarkers associated with obesity [228]. Dietary anthocyanins have also been found to have beneficial effects on chronic inflammatory disorders such as intestinal diseases [188]. The antioxidant, anti-inflammatory and immunomodulatory properties of anthocyanins contribute to their anti-inflammatory effects [229]. Basu et al. [196] showed that the consumption of two cups of low-energy cranberry juice per day significantly reduced lipid oxidation and increased the plasma antioxidant capacity in a group of women with MetS. In the study group consuming 50 g of freeze-dried blueberries, there was a significant reduction in plasma oxidised LDL and oxidative stress markers (malondialdehyde and hydroxynonenal) compared with the control group [196]. Several studies have reported significant reductions in C-reactive protein levels with anthocyanin supplementation: with the equivalent of 400 g of fresh bilberries (40 g of dried bilberries, equivalent to 200 g of fresh bilberries, and 200 g of bilberry purée) [224], following the administration of 300 mL/day blackberry juice with pulp [204] and among women who received 320 mg of anthocyanins [214]. In addition, bilberry consumption reduced the pro-inflammatory cytokines IL-6 and IL-12 [224]. However, Paquette et al. [207] and Xie et al. [208] did not find that bioactive compounds altered markers of inflammation and oxidative stress. Anthocyanins and phenolic acids from purple carrots also did not significantly reduce C-reactive protein levels [198]. Moreover, in a group of 60 people with abdominal obesity and elevated serum lipids, a 12-week intervention with 25 or 50 g of freeze-dried strawberries did not reduce C-reactive protein levels [199].

#### 5.3.4. Antioxidant Properties

Fruits and vegetables rich in anthocyanins show strong antioxidant properties. This is confirmed by studies with humans, indicating potential health benefits also in reducing the symptoms of metabolic syndrome. Due to their ability to potentially scavenge free radicals and reduce oxidative stress, the consumption of anthocyanins from a diet rich in fruit and vegetables has been linked to slowing the progression of oxidative damage [230]. Anthocyanins protect biological systems from free radical toxicity through various avenues and, as potent antioxidants, act as reducing factors in the electron transfer reaction pathway, transferring electrons to free radicals with unpaired electrons [231,232]. Anthocyanins interact with the NF-κB and AP-1 signal transduction pathways, which respond to oxidative signals, and with the Nrf2/ARE pathway and its regulated cytoprotective proteins (GST, NQO, HO-1), involved in cellular antioxidant defence as the elimination/inactivation of toxic compounds, thus counteracting oxidative stress-induced changes [218]. One study relying on the administration of 480 mL/day of cranberry juice to individuals with established MetS reported a significant increase in plasma antioxidant capacity (1.5 ± 0.6 to 2.2 ± 0.4 µmol/L) and a decrease in malondialdehyde concentration (3.4 ± 1.1 to 1.7 ± 0.7 µmol/L) [219]. Also, in a study by Kuntz et al. (2014), a reduction in malondialdehyde concentrations and an increase in total antioxidant activity as determined by the Trolox equivalent antioxidant capacity (TEAC) method was observed in plasma and urine after the consumption of 330 mL/day of anthocyanin-rich juice or smoothie [218].

#### 5.3.5. Influence on Body Composition and Measurements

Researchers have also assessed the effects of anthocyanins on body weight in humans. Numerous studies have shown that interventions with anthocyanins reduced BMI and body fat percentage [193,194,233,234]. In particular, anthocyanin supplementation at a dose of ≤300 mg/day for 4 weeks effectively reduced BMI and body weight [194]. However, Tiwari et al. [235] analysed 21 clinical trials and 27 preclinical studies and found that with a daily anthocyanin intake of ≤300 mg for 4 weeks, BMI reductions were observed more frequently in adults without obesity. During a 24-year follow-up of 124,086 healthcare workers in the United States, an increased intake of anthocyanins mainly from blueberries and strawberries was inversely correlated with weight gain [236]. An 8-week intervention in subjects with a BMI > 23 kg/m^2^ or a WC > 90 cm for men or >85 cm for women with anthocyanin-rich black soybean testa extracts had a beneficial effect on reducing WC and hip circumference [205]. Different results were obtained after a 4-week nutritional intervention involving the addition of anthocyanins and phenolic acids from purple carrots: there was no significant reduction in body mass or improvement in body composition [198]. In a group of women with overweight or obesity, the addition of 500 mL of commercial orange juice to the diet has no significant effect on body weight [206]. Moreover, a dose equivalent to 400 g of fresh bilberries for 8 weeks did not result in a significant weight reduction in the intervention group [224].

#### 5.3.6. Effects on BP

Anthocyanins are being investigated by researchers in terms of their potential effects on BP in humans, but the results of the studies are inconclusive. These compounds have been shown to have antihypertensive potential via the renin–angiotensin–aldosterone pathway based on molecular docking calculations. Anthocyanin-derived compounds such as delphinidin, petunidin, malvidin, cyanidin, peonidin and pelargonidin have potential as antihypertensive drugs [237]. Basu et al. [195] assessed the effects of anthocyanins on BP in a group of 48 individuals with obesity and MetS. They found that consuming two glasses of juice containing 50 g of freeze-dried blueberries per day could significantly reduce SBP and DBP [195]. Aghababaee et al. [200] also reported a significant decrease in SBP in the intervention group [200], while Novotny et al. [202] reported a significant decrease in DBP. In contrast, the results of other studies have demonstrated no significant effect of anthocyanins on BP regulation in humans [198,199,208,212,217,238,239].

## 6. Summary and Observations

The divergent results obtained by different research groups are most likely due to the use of different sources of anthocyanins; variations in doses, preparations and forms used in the experiments; the durations of the observations; and methodological differences [240,241,242]. In addition, anthocyanins are affected by gastrointestinal pH and ions, which impact the bioactivity and bioavailability of these polyphenolic compounds [243]. Compounds in food may act synergistically. More often than not, it is not possible to consider all of the nutrients contained in foods when conducting studies. Additionally, the use of extracts containing only anthocyanins may be more effective when assessing their effects on the human body [244]. Differences in the effects of anthocyanins on the human body may also be related to socio-cultural and ethnic characteristics, as well as the season and technical advances in the agri-food industry [240,245]. To our knowledge, there have been few studies conducted with large population groups to confirm the beneficial effects of anthocyanin intake. Most of the available research details pilot studies conducted mainly in small groups and in the short term (usually 8–12 weeks). There is a lack of studies determining the long-term impact of anthocyanin intake on the prevention of diet-related diseases, especially among children and adolescents. Additional research is needed on foods with functional properties and phytopharmaceuticals containing anthocyanins, with a focus on components with synergistic effects.

## 7. Conclusions

This review highlights the need for a deeper understanding of the mechanisms involved in the metabolism and bioavailability of anthocyanins found in food. It would also be worth exploring the impact of food-derived anthocyanins on MetS development. The current scientific evidence suggests that anthocyanins are an effective, widely available and inexpensive way to prevent and treat MetS and its complications. An increase in the consumption of anthocyanin-rich foods may contribute to the maintenance of normal body weight and modulation of the lipid profile in adults. This knowledge may contribute to revisions in dietary recommendations against obesity and cardiovascular diseases and their possible consequences. The role of anthocyanins in the global food chain should be understood and considered when making dietary choices. Hopefully, the present review will contribute to revising dietary recommendations to reduce the prevalence of MetS while improving the health and quality of life of individuals with MetS.

## Figures and Tables

**Table 1:** Diagnostic criteria for metabolic syndrome [23].

Necessary Condition for Diagnosis	
Diagnosed android obesity	WC > 102 cm (men) and >88 cm (women) or BMI ≥ 30 kg/m^2^
At least two of the following are required	
Disordered carbohydrate metabolism	FG ≥ 100 mg/dL or OGTT2h ≥ 140 mg/dL or HbA1c ≥ 5.7% or treatment started
Arterial hypertension	SBP ≥ 130 mmHg or DBP ≥ 85 mmHg or (home measurement): SBP ≥ 130 mmHg or DBP ≥ 80 mmHg or treatment started
Dyslipidaemia	Non-HDLc ≥ 130 mg/dL or use of hypolipidaemic treatment

Abbreviations: BMI, body mass index; DBP, diastolic blood pressure; FG, fasting glucose; HbA1c, glycated haemoglobin; non-HDLc, non-high-density lipoprotein cholesterol; OGTT2h, 2 h oral glucose tolerance test; SBP, systolic blood pressure; WC, waist circumference.

**Table 2:** Anthocyanin content of selected fruits and vegetables.

Fruit or Vegetable	Form	Anthocyanin Content	Source
Chokeberry	Fresh fruit	278.2–686.0 mg/100 g FW	[49,69,70,71]
	Pomace	1602.0–6280.0 mg/100 g DW	[68,72]
		171.1 mg/100 g	[73]
	Juice	38.8–1118.0 mg/L	[74,75]
		808.0–1527.0 mg CGE/L	[68]
	Syrup	188 mg/L	[75]
	Fruit tea	479.0–1557.0/100 g DW	[68]
	Extract	9360 mg/100 g	[55]
		233.5 mg/L	[60]
		14,733.5 mg/kg FW	[56]
Bilberry	Fresh fruit	0.34–0.47 mg/100 g	[76]
		2878 mg/100 g DW	[77]
		329.0 mg CGE/100 g FW	[78]
		0.041–0.166 μg/mL	[79]
	Extract	1.89–5.57%	[80]
Blackberry	Fresh fruit	48.1–122.8 mg/100 g FW	[81,82]
		79.59–465.4 mg CGE/100 g FW	[51]
	Frozen fruit	46.1–118.5 mg/100 g FW	[82]
	Dried fruit	113.08–3924.2 CGE/100 g DW	[83]
	Extract	5.3–4.5 mg/100 mL	[84]
Blackcurrant	Fresh fruit	62.8–287.8 mg CGE/100 g	[50,85]
		113.8 mg/100 g	[81]
	Lyophilised	163.1 mg/100 g DW	[86]
	Frozen fruit	183.0–446.0 mg CGE/100 g	[87]
	Juice	1529.0–2083.0 mg/L	[88]
	Pomace	55.1 mg/100 g	[89]
	Extract	86.0–340.0 mg/L	[90]
Blueberry	Fresh fruit	0.1–534.2 mg/100 g	[54,76,81,91,92]
	Dried powder	45,918.0 mg/100 g	[91]
	Juice	3909.0 mg/mL	[93]
	Extract	1301.2 mg/100 g FW	[56]
		5.8–11.4 mg/mL	[84]
Cherry	Fresh fruit	0.2–344.9 mg/100 g	[53,94]
		20.0–120.0 mg CGE/100 g	[95]
	Extract	117.2 mg/100 g FW	[56]
		19.9 mg CGE/100 g DW	[96]
Cranberry	Fresh fruit	44.6–835.2 mg/100 g	[48,97,98]
	Juice	0.398 mg/mL	[93]
	Extract	520.0 mg/100 g	[57]
Elderberry	Pomace	30,500 mg/100 g DW	[99]
	Juice	1090 mg/L	[100]
	Extract	443.6–1413.8 mg/100 g FW	[56,101]
Raspberry	Fresh fruit	37.5–2199.0 mg/100 g	[47,81,102,103]
		12.0–325.5 mg CGE/100 g FW	[51]
		9.3 mg/100 g of dry material	[104]
		97.3 μM/100 g	[105]
	Lyophilised	0.0–314.2 mg/100 g DW	[86]
	Juice powder	1.5–2.5 g/100 g	[106]
	Extract	67.1 mg/100 g	[56]
		2.3–3.7 mg/100 mL	[84]
Red currant	Fresh fruit	5.0–19.3 mg/100 g	[81,107]
		12.1–22.1 mg CGE/100 g	[50]
	Frozen fruit	10.7–28.4 mg/100 g	[107]
	Juice	5.8–25.6 mg/100 g	[107]
	Extract	1.8–6.8 g/L	[90]
Strawberry	Fresh fruit	31.0–189.0 mg/100 g FW	[61,62]
		145.8–204.3 mg/100 g DW	[52]
	Extract	16.6 mg/100 g FW	[56]
		38.0 mg CGE/100 g fruit	[108]
		0.7–1.1 mg/100 mL	[84]
Eggplant	Fresh vegetable	0.01–72.4 mg/100 g	[109]
	Frozen pulp	3.2–6.2 × 10^5^ mg Del-3-glc/100 g FW	[110]
	Peel	0.04–112.2 mg/100 g	[109]
	Frozen peel	1208.1– 84,652.0 mg Del-3-glc/100 g FW	[110]
	Extract	0.5–3.8 mg/100 mL	[84]
Red cabbage	Fresh vegetable	23.2–275.4 mg/100 g FW	[58,111]
		1111.0–1780.0 mg CGE/100 g DW	[112]
		16.0–889.0 μg/mL	[113]
	Extract	24.7–191.4 mg/100 g	[59]
		1.0–547.3 mg/L	[111,114,115,116]
		14.5–89.2 mg CGE/g FW	[117]
Red kidney bean	Fresh vegetable	8.1–10.5 mg/100 g	[118]
	Peel/seed coat	0.1–0.5 mg/g	[119]
		0.0–2.8 mg/g dried seed	[120]
	Extract	39.6–40.8 mg/100 g	[121]
Red onion	Fresh vegetable	28.7–103.0 μg/g FW	[122,123,124]
		1.6–1.8 mg CGE/g DW	[125]
	Extract	17.9 mg/L	[126]

Abbreviations: CGE, cyanidin-3-glucoside equivalents; Del-3-glc, delphinidin-3-glucoside equivalents; DW, dry weight; FW, fresh weight.

**Table 4:** The effects of anthocyanins on the components of metabolic syndrome (in vivo studies in animal models).

Ref.	Study Group	Daily Intake	Period of Consumption	Effect on the Body
[151]	Spontaneously hypertensive rats	Purple corn, purple sweet potato and red radish (1 mass% of diets)	15 weeks	↓ SBP and HR
[152]	Diet-induced obese mice	Norton grape pomace extract (250 mg/kg bw)	12 weeks	↓ hsCRP; no effect on oxidative stress
[153]	Hamsters	Mulberry aqueous extracts	12 weeks	↓ Body weight, visceral fat, TG, free fatty acids and hepatic lipids
[154]	Obese mice	Combination of mulberry leaf extract (MLE) and mulberry fruit extract (MFE): 133 mg/kg bw or 333 mg/kg bw MLE or 133 mg MLE and 67 mg MFE/kg bw or 333 mg MLE and 167 mg MFE/kg bw	12 weeks	↓ TG, liver lipid peroxidation and adipocyte size; improvement in hepatic steatosis, body weight gain, fasting plasma glucose and insulin, hsCRP, TNF-α and IL-1 in liver and adipose tissue
[155]	24 adult male Wistar rats	Honeysuckle berry extract (2 g/kg bw)	4 weeks	↑ Bacterial α-glucosidase and β-glucosidase activity; normalisation of plasma TG and insulin levels as well as insulin resistance
[156]	Obese Zucker rats	Diet with 8% wild blueberries	8 weeks	↓ TNF-α, IL-6 and hsCRP; ↑ adiponectin
[157]	Mice fed a high-fat diet	Honeysuckle anthocyanins (50, 100 or 200 mg/kg bw)	8 weeks	Honeysuckle anthocyanins at 100 or 200 mg/kg bw: suppression of body weight gain; ↓ serum and liver lipids, insulin and leptin; ↑ adiponectin
[158]	Mice fed a high-fat diet	Sweet potato	20 weeks	Improvement in fasting blood glucose as well as glucose and insulin tolerance; suppression of ROS; restoration of antioxidant enzyme activities
[159]	Rabbits fed 1% cholesterol	Cornelian cherry (100 mg/kg bw)	60 days	↓ Serum TG and proinflammatory cytokine levels; ↑ PPARα protein expression in the liver
[160]	18 male Wistar rats	25 mL of black chokeberry (*Aronia melanocarpa*) juice	90 days	↓ TC, LDLc and atherogenic risk
[161]	Sprague Dawley rats	100 or 300 mg/kg bw blackcurrant (*Ribes nigrum*)	8 weeks	Suppression of increased liver weight and epididymal fat weight, oral glucose tolerance and expression IRS-1 and *p*-AMPK in muscle; ↓ hsCRP, total bilirubin, leptin, insulin, TC, TG and LDLc
[162]	Rabbits fed 1% cholesterol	Loganic acid (20 mg/kg bw) or a mixture of anthocyanins (10 mg/kg bw)	60 days	Loganic acid or anthocyanin mixture: ↓ TC, LDLc, TG and ox-LDL in the plasma ↑ HDLc; loganic acid alone: ↓ TNF-α and IL-6; anthocyanin mixture alone: ↑ PPARγ and PPARα in the liver
[163]	60 mice	Cherry and mulberry anthocyanins (200 mg/kg bw)	8 weeks	↓ Body weight gain, serum glucose, leptin and TNFα and IL-6 expression; ↑ SOD and GSH-PX activities;
[164]	Obesity induced in C57BL/6 mice by feeding a high-fat diet	Black rice, black soybean or purple corn anthocyanins (200 mg/kg bw)	12 weeks	↓ body weight and TNF-α and IL-6 expression; ↑ faecal butyric acid levels and SOD and GSH-PX activities
[165]	Spontaneously hypertensive rats	Normal diet with 10% freeze-dried aronia berries	28 days	Inhibition of the kidney renin–angiotensin system; ↓ BP; no effect on body weight
[166]	Wistar rats	Cranberry extract (200 mg/kg bw)	30 days	↓ Body mass gain, TG, corticosterone, hepatic cholesterol and fatty acid synthase; ↓ lipid peroxidation, protein carbonylation (liver and adipose tissue) and accumulation of fat in the liver
[167]	Streptozotocin-induced diabetic rats	Red cabbage extract (800 mg/kg bw)	4 weeks	↓ Blood glucose; ↓ glycated and foetal Haemoglobin; improvement in glucose tolerance; ↑ serum insulin, proinsulin C-peptide and the number of pancreatic β-cells
[168]	High-cholesterol-diet-induced hypercholesterolaemic mice	Extract with lingonberry anthocyanins	10 weeks	↓ Body weight, daily food intake, liver weight, visceral adipose content, TC, LDLc and fasting blood glucose; ↑ HDLc
[169]	C57BL/6 mice	Raspberry anthocyanins (200 mg/kg bw)	12 weeks	↓ Body weight gain and TNFα and IL-6 expression; ↑ faecal butyric acid levels and SOD and GSH-PX activities
[170]	Zucker diabetic fatty rats	Cornelian cherry (500 or 1000 mg/kg bw)	10 weeks	Both doses: no effect on HOMA-IR; 1000 mg/kg bw: ↓ glucose
[171]	Rat model of type 1 diabetes mellitus	Cornelian cherry (20 mg/kg bw)	14 days	↓ Blood glucose; ↑ reduced glutathione; improvement in glucose tolerance
[172]	Hyperglycaemic rats	Purple sweet potatoes (50 or 100 mg/kg bw)	35 days	↓ MDA in the blood, liver and kidney; urea and creatinine levels; serum glutamate oxalacetate transaminase and glutamate pyruvate transaminase levels
[173]	Streptozotocin-induced diabetic rats	Purple potato (the Blue Congo variety) extract	2 weeks	↓ Blood glucose; improvement in glucose tolerance; ↓ glycated haemoglobin and MDA; reinstatement of antioxidant enzyme activities
[174]	C57BL/6J mice fed a high-fat diet	Black rice anthocyanins	14 weeks	↓ Body weight gain, TG, TC, steatosis scores and insulin resistance index
[175]	C57BL/6J mice fed a high-fat diet	Blackcurrant anthocyanins	12 weeks	Alleviated high-fat-diet-induced obesity, hyperlipaemia and hepatic steatosis; improvement in hepatic lipid metabolism
[176]	C57BL/6J mice fed a high-fat diet	Pure water containing 0.8% of a crude extract of anthocyanins from *Lycium ruthenicum* fruit	14 weeks	↓ Body weight, TC and LDLc; inhibition of lipid accumulation in liver and white adipose tissue and pancreatic lipase activity; regulation of the intestinal microbiota
[177]	Streptozotocin- induced diabetic mice	100 and 400 mg/kg blueberry anthocyanin extracts	5 weeks	↓ Body weight, blood and urine glucose, TG and TC; ↑ AMPK activity
[178]	Wistar rats	(50, 100 or 200 mg/kg bw)	4 weeks	↓ SBP, DBP, MABP and HR
[179]	Sprague Dawley rats	Infusion of anthocyanins (10 mg/kg bw, 0.4 μL/h)	4 weeks	↓ Caspase-1, IL-1β, TNF-α, ROS and BP
[180]	Streptozotocin- induced diabetic mice	Black bean peel extract (containing 40% anthocyanin, 400 mg/kg bw)	4 weeks	↑ T-AOC; ↓GSH
[181]	C57BL/6J mice	100, 400, or 800 mg/kg bw blueberry anthocyanin extract	0.1–12 h	↑ T-AOC; ↓ MDA
[182]	Wistar rats	100, 200, 300 or 400 mg/kg bw of anthocyanin extract	4 weeks	↓ CAT, MDA, GSH-PX, SOD
[183]	Dankin Hartley guinea pigs	Diet with 8% blueberries	75 days	↓ TG, MDA

Abbreviations: BP, blood pressure; bw, body weight; CAT, catalase; GSH, glutathione; GSH-PX, glutathione peroxidase; HDLc, high-density lipoprotein cholesterol; HOMA-IR, homeostatic model assessment for insulin resistance; HR, heart rate; hsCRP, high-sensitivity C-reactive protein; IL-1, interleukin-1; IL-6, interleukin-6; IRS-1, insulin receptor substrate-1; LDLc, low-density lipoprotein cholesterol; MABP, mean arterial blood pressure; MDA, malondialdehyde; ox-LDL, oxidised low-density lipoprotein; *p*-AMPK, phosphorylated AMP-activated protein kinase; PPARα, peroxisome proliferator-activated receptor α; PPARγ, peroxisome proliferator-activated receptor γ; ROS, reactive oxygen species; SBP, systolic blood pressure; SOD, superoxide dismutase; T-AOC, total antioxidative capability; TC, total cholesterol; TG, triglycerides; TNF-α, tumour necrosis factor α; ↓, lowering the concentration; ↑, increase the concentration.

**Table 5:** The effects of anthocyanins and their supplementation on the components of metabolic syndrome (in vivo studies with humans).

Ref.	Study Group	Daily Intake	Period of Consumption	Effect on the Body
[195]	48 people with MetS	480 mL of water with vanilla extract and 50 g of freeze-dried blueberries	8 weeks	↓ SBP, DBP, oxidised LDLc, MDA and hydroxynonenal; no effect on glucose levels and the lipid profile
[196]	36 people with MetS	480 mL of cranberry juice	8 weeks	↑ Plasma antioxidant capacity; ↓ oxidised LDL and malondialdehyde
[197]	40 children with dyslipidaemia (9–16 years old)	50 g of *Cornus mas* L. fruit	6 weeks	↑ HDLc and apo A-I; ↓TC, LDLc, TG and apo B
[198]	16 women with obesity and normal lipid and inflammatory marker levels	Dried purple carrot delivering 118.5 mg/day of anthocyanins and 259.2 mg/day of phenolic acids	4 weeks	↓ HDLc; no effect on body mass, body composition, TC, LDLc, BP and CRP
[199]	60 people with abdominal adiposity and elevated serum lipids	25 or 50 g of freeze-dried strawberries reconstituted in 2 cups (474 mL) of water	12 weeks	↓ TC and LDLc; no effect on measures of adiposity, BP, glycaemia, HDLc, TG and CRP
[200]	72 people with dyslipidaemia	300 mL/day of blackberry juice with pulp	8 weeks	↑ HDLc and SBP; ↓ hsCRP
[201]	58 people with diabetes mellitus	Supplementation with 320 mg of anthocyanins	24 weeks	↑ HDLc and TRAP; ↓ LDLc, TG, HOMA-IR, IL-6 and TNF-α
[202]	56 people with a BMI of 20–38 kg/m^2^ and in basic good health	240 mL of low-calorie cranberry juice	8 weeks	↓ TG, CRP, DBP, FBG and HOMA-IR
[203]	74 people with NAFLD	Purified anthocyanins (320 mg/day) from bilberry and blackcurrant	12 weeks	↓ FBG and HOMA-IR
[204]	36 apparently healthy people (25 men and 11 women)	150 g of frozen bilberries (*Vaccinium myrtillus* L.) 3 times per week	6 weeks	Men and women: ↑ HDLc; ↓ TG, glucose, albumin, aspartate aminotransferase and γ-glutamyltransferase; men only: ↑ LDLc; women only: ↓ LDLc
[205]	63 people with BMI >23 kg/m^2^ or WC > 90 cm (for men) or >85 cm (for women)	2.5 g/day of black soybean testa extracts	8 weeks	↓ WC; hip circumference; TG; LDLc; non-HDLc; and arteriosclerosis indicators such as (TC)/HDLc and LDLc/HDLc
[206]	11 women with overweight or obesity	500 mL of commercial pasteurised red orange juice (250 mg of anthocyanins/day)	12 weeks	↓ TC and LDLc; no effect on weight loss
[207]	41 people with overweight or obesity (BMI ≥ 25 kg/m^2^) with insulin resistance (fasting plasma insulin level >60 pmol/L)	Beverage with 1.84 g of a mixture of dry strawberry (*Fragaria* × *ananassa* Duch) and cranberry (*Vaccinium macrocarpon* L.) (333 mg of polyphenol)	6 weeks	↑ Insulin sensitivity based on the hyperinsulinaemic–euglycaemic clamp and C-peptide; no effect on the lipid profile and markers of inflammation and oxidative stress
[208]	49 healthy adult former smokers	500 mg of chokeberry extract	12 weeks	↓ TC, LDLc; no effect on BP and biomarkers of inflammation and oxidative stress
[209]	27 men with overweight or obesity (BMI > 25 kg/m^2^)	High-fat diet (40% of energy from fat) that contained 600 g/day blackberries (~1476 mg of flavonoids and ~361 mg of total anthocyanins per day)	1 week	↓ iAUC for insulin; ↑ AUC for NEFAs
[210]	115 adults with overweight or obesity (BMI ≥ 25 kg/m^2^)	0.5 or 1 cup blueberries/day (182 or 364 mg of anthocyanins and 439 or 879 mg phenolics, respectively)	6 weeks	↑ HDLc; no effect on TC, LDLc, HOMA-IR, HbA1c and BP
[211]	50 healthy people (36 women and 14 men)	300 mL/day of a 50%/50% mixture of berry and apple juice	21 days	Men and women: ↑ HDLc and total antioxidant status; men only: ↓ TC and LDLc
[212]	84 people ‘at cardiovascular risk’	100 mL/day of chokeberry juice with a high or low dose of polyphenols (177.11 or 294.28 mg total polyphenols and 113.3 mg/100 mL or 28.3 mg/100 mL total cyanidin-3-glucoside equivalents, respectively)	4 weeks	↑ SFAs; *↓* PUFAs; no effect on TC, LDLc, SBP and DBP
[213]	25 people with overweight (BMI > 25 kg/m^2^)	New Zealand blackcurrant extract (600 mg/day)	8 days	↓ hsCRP; improvement in circulating insulin
[214]	55 people with MetS	Supplementation with 320 mg of anthocyanins	4 weeks	Men and women: ↓ FBG, TG and LDLc; women only: ↓ CRP; no effect on uric acid and HDLc
[215]	35 women and men (with MetS or healthy)	Veg-encapsulated anthocyanin extract taken two times a day (320 mg of anthocyanins)	4 weeks	↑ PPAR-γ and SOD gene expression; ↓ hsCRP and the expression of NF-κB-dependent genes (TNF-α, IL-6 and IL-1α)
[216]	18 people with NAFLD	20 mL/day of cornelian cherry fruit extract as liquid form (32 mg total anthocyanins)	12 weeks	No effect on LAP, AIP, CRI and AC
[217]	50 people with type 2 diabetes mellitus or hypertension	Two 300 mg capsules of anthocyanin extract (total of 600 mg/day)	30 days	↓ hsCRP; no effect on BP and blood glucose levels
[218]	36 adults with features of metabolic syndrome	480 mL cranberry juice (24 mg total anthocyanins)	8 weeks	↓ ox-LDL and MDA; ↑ antioxidant capacity in plasma; no effect on biomarkers of inflammation, glucose and lipids
[219]	30 healthy females	330 mL juice or smoothie (total phenolics: juice—3227; smoothie—3435 mg/L)	14 days	↓ MDA; no effect on IL-2, IL-6, IL-8 and IL-10, CRP, TNF-α, SOD and GSH-PX

Abbreviations: AC, atherogenic coefficient; AIP, atherogenic index of plasma; apo A-I, apolipoprotein A-I; apo B, apolipoprotein B; AUC, area under the curve; BMI, body mass index; BP, blood pressure; CRI, Castelli risk index I; CRP, C-reactive protein; DBP, diastolic blood pressure; FBG, fasting blood glucose; HbA1c, glycated haemoglobin; HDLc, high-density lipoprotein cholesterol; HOMA-IR, homeostatic model assessment for insulin resistance; hsCRP, high-sensitivity C-reactive protein; iAUC, incremental area under the curve; IL-1α, interleukin 1α; IL-6, interleukin-6; LAP, lipid accumulation product; LDLc, low-density lipoprotein cholesterol; MDA, malondialdehyde; MetS, metabolic syndrome; NAFLD, non-alcoholic fatty liver disease; NEFAs, non-esterified fatty acids; non-HDLc, non-high-density lipoprotein cholesterol; ox-LDL, oxidised low-density lipoprotein; PPARγ, peroxisome proliferator-activated receptor γ; PUFAs, polyunsaturated fatty acids; SBP, systolic blood pressure; SFAs, saturated fatty acids; SOD, superoxide dismutase; TC, total cholesterol; TGs, triglycerides; TNF-α, tumour necrosis factor α; TRAP, tartrate-resistant acid phosphatase; WC, waist circumference; ↓, lowering the concentration; ↑, increase the concentration.
